# EMT: Present and future in clinical oncology

**DOI:** 10.1002/1878-0261.12091

**Published:** 2017-06-27

**Authors:** Patricia G. Santamaria, Gema Moreno‐Bueno, Francisco Portillo, Amparo Cano

**Affiliations:** ^1^ Departamento de Bioquímica Instituto de Investigaciones Biomédicas ‘Alberto Sols’ (CSIC‐UAM) Universidad Autónoma de Madrid (UAM) IdiPAZ CIBERONC Madrid Spain; ^2^ Fundación MD Anderson International Madrid Spain

**Keywords:** clinical trials, EMT, MET, metastasis, preclinical models, therapy resistance

## Abstract

Epithelial/mesenchymal transition (EMT) has emerged as a key regulator of metastasis by facilitating tumor cell invasion and dissemination to distant organs. Recent evidences support that the reverse mesenchymal/epithelial transition (MET) is required for metastatic outgrowth; moreover, the existence of hybrid epithelial/mesenchymal (E/M) phenotypes is increasingly being reported in different tumor contexts. The accumulated data strongly support that plasticity between epithelial and mesenchymal states underlies the dissemination and metastatic potential of carcinoma cells. However, the translation into the clinics of EMT and epithelial plasticity processes presents enormous challenges and still remains a controversial issue. In this review, we will evaluate current evidences for translational applicability of EMT and depict an overview of the most recent EMT *in vivo* models, EMT marker analyses in human samples as well as potential EMT therapeutic approaches and ongoing clinical trials. We foresee that standardized analyses of EMT markers in solid and liquid tumor biopsies in addition to innovative tools targeting the E/M states will become promising strategies for future translation to the clinical setting.

**Abbreviations**
CFP: cyan fluorescent protein
CRC: colorectal cancer
CTC: circulating tumor cell
eDTCs: early disseminated tumor cells
EMT: epithelial/mesenchymal transition
EMT‐TF: epithelial/mesenchymal transition transcription factor
EpCAM: epithelial cell adhesion molecule
FSP1: fibroblast‐specific protein 1
GFP: green fluorescent protein
HCC: hepatocellular carcinoma
HER2/Neu/ERBB2: ERBB2 receptor tyrosine kinase 2
HNSCC: head and neck squamous cell carcinoma
ILK: integrin‐linked kinase
MET: mesenchymal/epithelial transition
MMP: metalloproteinase
NCID: Notch intracellular cytoplasmic domain
NSCLC: non‐small‐cell lung cancer
PCa: prostate cancer
PDAC: pancreatic ductal adenocarcinoma
RFP: red fluorescent protein
SCC: squamous cell carcinoma
TCGA: The Cancer Genome Atlas
TGF‐β: tumor growth factor beta
WNT: wingless
YFP: yellow fluorescent protein

## 1 Introduction

### 1.1 Epithelial plasticity during carcinoma progression and metastasis

Since the beginning of the present century, a plethora of *in vitro* studies have firmly established that activation of the EMT program promotes tumor cell invasion and metastasis and have defined the molecular players, environmental cues, and signaling pathways implicated in EMT induction (Chaffer *et al*., 2016; De Craene and Berx, 2013; Lambert *et al*., 2017; Lamouille *et al*., 2014; Nieto, 2013; Nieto and Cano, 2012; Nieto *et al*., 2016; Peinado *et al*., 2007; Thiery, 2002; Thiery *et al*., 2009; Yang and Weinberg, 2008). EMT is envisioned as the loss of epithelial status and apico‐basal polarity sustained on cell–cell adhesion molecules in order to gain mesenchymal traits. This transition entails the up‐ and downregulation of different proteins responsible for a profound cellular reorganization resulting in the acquisition of enhanced migratory and invasive properties (Nieto *et al*., 2016; Thiery *et al*., 2009). Context‐dependent signaling transduction pathways and microenvironmental signals, such as hypoxia, oxidative stress, nutrient deprivation, or inflammation, impinge on particular EMT transcription factors (EMT‐TFs) such as Snail1/Snail2, ZEB1/ZEB2, and Twist1, responsible to induce and sustain the mesenchymal phenotype (De Craene and Berx, 2013; Nieto and Cano, 2012; Peinado *et al*., 2007). The EMT process is tightly controlled in normal tissues through a complex regulation of EMT‐TFs, with interconnected regulatory networks operating at different transcriptional and post‐translational levels (i.e., alternative splicing, noncoding RNAs, epigenetic regulatory mechanisms, protein stability) (De Craene and Berx, 2013; Diaz‐Lopez *et al*., 2014; Nieto, 2011; Nieto and Cano, 2012; Peinado *et al*., 2007; Tam and Weinberg, 2013; Yang and Weinberg, 2008). On the other hand, EMT‐TFs control gene expression programs that are far beyond the acquisition of mesenchymal traits and influence multiple cellular processes through the direct or indirect transcriptional regulation of numerous genes (Nieto and Cano, 2012; Nieto *et al*., 2016; Thiery *et al*., 2009). The pleiotropic effects of the EMT program, when activated in tumor cells, favor the acquisition of a compendium of cellular abilities intimately linked to tumor progression and metastasis besides influencing tumor evolution and response to therapeutic treatments (Lambert *et al*., 2017; Nieto *et al*., 2016). Furthermore, the EMT process within the tumor context is highly dynamic, implying transient and reversible states, thus resembling embryonic development where multiple rounds of EMT and the reverse mesenchymal/epithelial transition (MET) processes occur as necessary steps for early embryogenesis and morphogenesis (Nieto, 2013; Nieto *et al*., 2016; Thiery *et al*., 2009). This reversibility might be indeed an essential feature of the metastatic cascade. As repeatedly appreciated by pathologists, distant metastases arising from carcinomas usually present the same histology as the primary tumor, indicating the maintenance or reacquisition of the epithelial morphology by disseminated tumor cells at distant sites (Brabletz, 2012a; Brabletz *et al*., 2005). In fact, the requirement of MET for the establishment of macrometastases was promptly enunciated at the emergence of the EMT field (Thiery, 2002), and supporting evidence has been recently provided (Korpal *et al*., 2011; Ocaña *et al*., 2012; Stankic *et al*., 2013; Tsai *et al*., 2012).

One of the misconceptions, widely extended in the field until recently, is that EMT implies a complete transdifferentiation from a functional epithelial cell into a mesenchymal‐like cell. However, numerous reports suggest that complete EMT is restricted to some types of tumors (i.e., carcinosarcomas) and that intermediate EMT states coexist in most tumors, with cells acquiring some mesenchymal properties without undergoing a full EMT (Nieto and Cano, 2012). Recent observations support that EMT and MET processes represent two final endpoint states of dynamic epithelial plasticity routes. Thus, intermediate states between epithelial (E) and mesenchymal (M) phenotypes might occur at different steps of the metastatic cascade with cells transitioning through hybrid E/M states during tumor dissemination (Chaffer *et al*., 2016; Diepenbruck and Christofori, 2016; Lambert *et al*., 2017; Nieto *et al*., 2016; Yeung and Yang, 2017).

Despite the overwhelming information accumulated on EMT and cell plasticity in tumor biology, at least two key questions are still unresolved: (a) the requirement of EMT/MET processes for metastasis to occur *in vivo* and (b) the actual relevance of EMT in the clinical practice. In the following sections, we provide an update of the evidence on the first issue and discuss recent insights into EMT translational opportunities presently and in the near future.

## 2 Epithelial plasticity is required for metastasis: lessons from mouse cancer models

**Figure 1:**
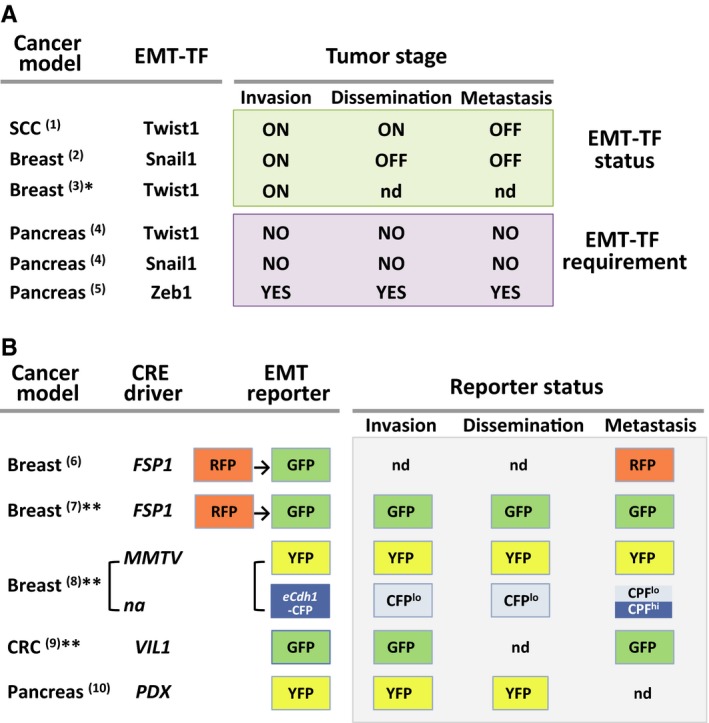
Genetic mouse models to evaluate the relevance of EMT in the metastatic process. Three steps of the metastatic cascade (invasion, dissemination, and distant metastasis) are selected. (A) Cancer mouse models based on knock‐out (KO) and/or knock‐in (KI) of specific EMT‐Ts. ^(1)^Twist1 conditional KO/KI (Tsai *et al*., 2012); ^(2)^Snail1 conditional KO/KI and Snail reporter (Tran *et al*., 2014); ^(3)^Snail1 transgene in the context of H‐Ras activation (Morel *et al*., 2012); ^(4)^Snail1 or Twist1 conditional KO (Zheng *et al*., 2015); ^(5)^ ZEB1 conditional KO (Krebs *et al*., 2017). The status (upper) or requirement (bottom) of the targeted EMT‐TFs in different metastatic steps is indicated. ON (activation), OFF (inactivation). (B) EMT lineage tracing models based on the activation and/or switching of fluorescent reporters (RFP, GFP, YFP, CFP) driven from the indicated promoters (*FSP1*,*MMTV*,*VIL1*,*PDX, Cdh1*) in different cancer mouse models. Breast cancer: ^(6)^(Fischer *et al*., 2015), ^(7)^(Zhao *et al*., 2016), ^(8)^(Beerling *et al*., 2016); CRC: ^(9)^(Chanrion *et al*., 2014); pancreatic cancer: ^(10)^(Rhim *et al*., 2012). The occurrence of EMT in each step is indicated by the color code of each of the fluorescent reporter. *FSP1*: fibroblast‐specific protein 1; *MMTV*, mouse mammary tumor virus; *Cdh1,* E‐cadherin; *VIL1*, villin 1; *PDX*, pancreatic duodenal homeobox 1. *HRas^G12D^ background; **Intravital imaging combined with reporter tracing. nd, not determined; na, not applicable.

To outline the *in vivo* functional contribution of EMT/MET processes to cancer progression, we will discuss the recent, and sometimes controversial, evidences obtained using genetically engineered mouse cancer models. Most of the current genetic models are based on the manipulation to knock‐out/knock‐in key regulators of EMT (i.e., EMT‐TF genes) and/or in EMT lineage tracing models (Fig. 1).

### 2.1 Mouse models of EMT‐TFs

Regarding genetic modulation of EMT‐TF genes, two recent reports used Snail1 or Twist1 to control EMT in breast or squamous cell carcinoma (SCC) cancer models, respectively (Tran *et al*., 2014; Tsai *et al*., 2012) (Fig. 1A). In the first study, the authors characterized several mouse models of breast cancer engineered to express an inducible Snail1 transgene in combination with endogenous Snail1 reporter and conditional Snail1 knockout. Their results supported that endogenous expression of Snail1 was restricted to primary tumors and was required and sufficient for breast cancer metastasis. Additionally, the authors showed that Snail1 expression during breast cancer metastasis was transient and they hypothesized that Snail1 continuous expression blocked the MET process required for metastasis to grow (Tran *et al*., 2014). In line with these findings, upregulation of Twist1 expression in a spontaneous SCC mouse model promoted invasive carcinoma progression and dissemination into the bloodstream (Tsai *et al*., 2012). Importantly, the authors show that Twist1 silencing at distant sites allowed the reversion of EMT to MET, which was a requisite for disseminated tumor cells to grow and form detectable metastases (Tsai *et al*., 2012). These data are consistent with other *in vitro* studies in breast cancer cell lines in which silencing of both Twist1 and PRRX1, another EMT‐TF, was required for efficient metastatic outgrowth at distant sites (Ocaña *et al*., 2012).

Although the above‐mentioned studies support an important role for dynamic EMT/MET in the development of metastasis *in vivo*, other recent reports controversially suggest that EMT is dispensable for invasion and metastasis but key to chemoresistance (Fischer *et al*., 2015; Zheng *et al*., 2015). Using genetic mouse models of pancreatic cancer reproducing the course of human pancreatic tumors, Zheng *et al*. (2015) reported that genetic suppression of Snail1 or Twist1 in primary pancreatic ductal adenocarcinomas (PDACs) did not alter tumor progression or metastatic outgrowth (Fig. 1A), thus concluding that EMT was dispensable for metastasis. A similar conclusion was derived from an EMT lineage tracing system in breast cancer models (Fischer *et al*., 2015; see below). One likely explanation for the unanticipated results obtained by Zheng *et al*. (2015) is that the deletion of Snail1 or Twist1 might be compensated by other EMT‐TFs as has been described in cell culture models (Diaz‐Lopez *et al*., 2015) or, alternatively, that EMT‐TFs expression is tumor specific, being Snail1 or Twist1 dispensable for pancreatic cancer metastasis. In support of the latter hypothesis, ZEB1 knock‐out in the mouse model of pancreatic cancer reduces the metastatic burden to about 30% without affecting the expression of Snail1 or other EMT‐TFs (Krebs *et al*., 2017). Interestingly, this study also showed that the deletion of ZEB1 freezes or halts pancreatic tumor cells in an epithelial state in which invasion, stemness, and metastatic colonization are dramatically suppressed, being cells in addition unresponsive to EMT‐inducing signals. This report thus emphasizes the nonredundant actions of different EMT‐TFs in metastasis generation *in vivo*. Although not directly addressing the metastatic potential, another transgenic breast cancer model based on the overexpression of Twist1 in the context of H‐Ras activation led to highly undifferentiated invasive tumors (Fig. 1A) with a claudin‐low phenotype (Morel *et al*., 2012) that exhibited intrinsic EMT features (Taube *et al*., 2010). The specific action of EMT‐TFs and their context dependence are also supported by lineage tracing experiments to evaluate tumor initiation in different genetically engineered knock‐in reporter breast cancer mouse lines, which demonstrated that the EMT program mediated by Snail2 (Slug) is required for normal mammary stem cells, while Snail1‐induced EMT accounts for the acquisition of stemness and tumor‐initiating properties of neoplastic lesions (Ye *et al*., 2015).

### 2.2 Lineage tracing mouse models

An additional concern regarding some mouse models genetically modified for EMT‐TFs expression is that they rely on the alteration of a particular EMT‐TF, underestimating the contribution of epithelial plasticity and hybrid E/M states to the metastatic cascade. To overcome this issue, several mouse lineage tracing models have been developed to follow the fate of epithelial cells during *in vivo* tumor progression without disturbing EMT‐TFs expression to better evaluate the requirement of EMT for metastasis (Fig. 1B).

One of the first animal model described, based on the mouse model of PDAC, allowed the detection of migrating and invading tumor cells by the yellow fluorescent protein (YFP) tracer (Rhim *et al*., 2012). Migrating tumor cells (YPF^+^) were detected already in initial premalignant pancreatic intraepithelial neoplasia (PanIN) lesions, presenting features of undergoing EMT (E‐cadherin^‐^/N‐cadherin^+^) and, noticeably, showing high ZEB1 expression and being able to disseminate in the bloodstream (Fig. 1B). Interestingly, isolated YPF^+^/E‐cadherin^‐^ cells from PanIN lesions displayed tumor‐initiating capacity and generated heterogeneous tumors containing E‐cadherin^‐^ and E‐cadherin^+^ cells (Rhim *et al*., 2012). This study supports that the EMT program occurs early in pancreatic tumor dissemination and that plasticity between epithelial and mesenchymal states during tumor development indeed arises *in vivo*; however, it did not address whether early disseminated cells have metastatic initiating abilities.

A similar approach was used in a mouse model for intestinal tumors conditionally activating the NOTCH receptor (NCID‐GFP: NOTCH cytoplasmic intracellular domain‐green fluorescent protein) and inactivating p53 within the intestine (Fig. 1B) (Chanrion *et al*., 2014). This model recapitulates many features of aggressive colorectal cancer (CRC), including lymph node and liver metastasis in addition to peritoneal carcinomatosis. GFP^+^ cells showing a gradient of epithelial to mesenchymal phenotypes were detected at the invasive and desmoplastic regions, indicating the plasticity of tumor cells during invasion. Expression of several EMT‐TFs such as Snail1, Snail2, Twist1, and ZEB1 was found in the desmoplastic area, while ZEB1 expression could be seen in the nuclei of invasive GFP^+^ cells that have undergone a partial or complete EMT, thus confirming previous observations on ZEB1 expression at the invasive front of human CRC samples (Spaderna *et al*., 2006). Results from *in vivo* GFP^+^ tumor cell lineage tracing and *ex vivo* imaging analyses of NCID/p53 tumor sections by two‐photon microscopy allowed the identification of individual mesenchymal‐like GFP^+^ cells as well as clusters of GFP^+^ cells at the invasive regions. Although mesenchymal‐like GFP^+^ cells were also detected at metastatic sites, the contribution of single EMT‐like cells migrated from the primary tumor could not be traced using the NCID/p53 mouse model. Noticeably, examination of human CRC samples revealed that activation of NOTCH in the context of p53 downregulation is significantly associated with metastatic CRC, supporting the validity of this NCID/p53 genetic model for further studies on epithelial plasticity (Chanrion *et al*., 2014).

Other novel EMT lineage tracing studies derive from breast cancer transgenic mouse models. Transgenic *MMTV‐PyMT* or *MMTV‐Neu* mice were engineered to express Cre recombinase in cells of mesenchymal lineage (*Fsp1*‐Cre) and a constitutive Cre‐switchable fluorescent marker (*lox‐RFP‐STOP‐lox‐GFP*). Thus, RFP^+^ (red fluorescent protein^+^) epithelial tumor cells undergoing a full EMT permanently convert into GFP^+^ cells (Fischer *et al*., 2015) (Fig. 1B). The authors showed that only a small proportion of tumor cells underwent EMT, whereas lung metastases were mainly composed of non‐EMT/RFP^+^ cells that maintained their epithelial phenotype, concluding therefore that EMT is dispensable for breast cancer metastasis. Besides, the authors also uncovered that EMT cells were responsible for recurrent lung metastasis after chemotherapy (Fischer *et al*., 2015), in line with the results obtained using the pancreatic cancer model discussed above (Zheng *et al*., 2015). The major limitation of the models generated by Fischer *et al*. (2015) is that they allow the detection of cells undergoing a full EMT, due to the restricted expression of the *Fsp1* (fibroblast‐specific protein 1) promoter to mesenchymal cells (Bhowmick *et al*., 2004), but might underestimate the detection of intermediate E/M states during the course of tumor cell dissemination. The ubiquitous stromal expression of GFP imposes an additional limitation for the accurate detection of the low population of GFP^+^ tumor cells *in vivo*, leaving open the question of their potential implication in the metastatic cascade.

An additional study used a similar lineage tracing model system allowing the tracking of converted RFP^+^ to GFP^+^ cells upon the activation of the *Fsp1* promoter in the context of *MMTV‐PyMT* breast cancer mouse model (Fig. 1B) (Zhao *et al*., 2016). Upon RFP^+^ cell isolation and orthotopic injection, the authors monitored EMT through the detection of GFP^+^ cells during tumor formation combining intravital imaging of live mice and tumor sections. RFP^+^ cells transforming to GFP^+^ mesenchymal‐like cells were detected and localized preferentially to blood vessels. These data clearly demonstrate that EMT indeed occurs in cells migrating from primary PyMT tumors; however, the use of the *Fsp1* promoter may favor the detection of tumor cells primed to acquire a full mesenchymal phenotype but precluding the detection of tumor cells in dynamic plastic states as those undergoing intermediate E/M transitions. This limitation was partially overcome in another approach also based on the breast cancer *MMTV*‐*PyMT* mouse model. This study combined the analysis of YFP^+^ tumor cells and endogenous E‐cadherin fused to mCFP (mouse cyan fluorescent protein) (E‐cadherin‐CFP^+^)‐expressing cells (Fig. 1B) (Beerling *et al*., 2016). Cell lineage tracing with high‐resolution intravital imaging allowed the detection of a small population of cells undergoing partial EMT (defined as E‐cadherin‐low: Ecad^lo^ or CPF^lo^). Importantly, the Ecad^lo^ population had the abilities to invade, migrate, intravasate, and extravasate besides reaching metastatic organs such as the liver. Rapid conversion of Ecad^lo^ cells to Ecad^hi^ (E‐cadherin‐high and thus epithelial) occurred at metastatic sites as early as upon two cell divisions (Fig. 1B), supporting the high plasticity potential of tumor cells at distant organs. Noticeably, a rare population of Ecad^lo^ cells could be detected in human breast tumors expressing a gene signature similar to that defined for mouse Ecad^lo^ cells. This study supports both the existence of EMT and epithelial plasticity *in vivo* and, remarkably, the interconversion between mesenchymal and epithelial states as soon as tumor cells reach the metastatic organ (Beerling *et al*., 2016).

Moreover, detection of early disseminated tumor cells (eDTCs) suffering a partial EMT (Ecad^lo^/Twist^hi^) was recently reported using the *MMTV*‐*Her2* breast cancer mouse model (Harper *et al*., 2016). Interestingly, the partial EMT detected in eDTC Her2^+^ cells seemed mediated by canonical WNT signaling and was associated with the inhibition of MAPK p38 activity promoting eDTCs survival during dissemination. In addition, eDTCs were even more invasive than primary tumor cells and able to reach distant metastatic sites where they remained dormant and eventually generated metastasis. Intriguingly also, mammospheres obtained from eDTCs exhibited higher metastatic potential than those derived from the bulk of the primary tumor, suggesting that their higher intrinsic plasticity conferred advantages for metastatic growth (Harper *et al*., 2016). However, whether the metastatic outgrowth of eDTCs required the shutdown of Twist1 expression and reacquisition of an epithelial phenotype (i.e., a MET process) is not yet clarified. This study, together with the previous report on pancreatic cancer models (Rhim *et al*., 2012), strongly supports the existence of EMT and plasticity processes *in vivo* at very early stages of tumor progression, even though their translation to human tumors is yet unclear. As recently discussed by others (Gomis and Gawrzak, 2016; Lambert *et al*., 2017), how disseminated tumor cells from early lesions acquire the additional genetic and epigenetic changes, including microenvironment adaptation, necessary for leaving dormancy and promoting metastasis outgrowth, is still far from understanding.

### 2.3 Models of intermediate E/M states

On the other hand, the emerging concept that intermediate or hybrid E/M states are more relevant to metastasis than fixed epithelial or mesenchymal phenotypes suggests that such a hybrid E/M status confers a transient metastable phenotype. Thus, the hybrid E/M status would facilitate transitions between different stages of epithelial plasticity and allow rapid adaptation and response to diverse environmental cues (Chaffer *et al*., 2016; Diepenbruck and Christofori, 2016; Nieto *et al*., 2016). Although a clear evidence for this premise is still lacking, recent works suggest the existence of phenotypic stability factors, such as OVOL (ovo‐like zinc finger) and GRHL2 (grainyhead‐like transcription factor 2), that can contribute to stabilize the hybrid E/M phenotype *in vivo* during mammary branch morphogenesis as well as in cancer (Jia *et al*., 2015; Jolly *et al*., 2016; Watanabe *et al*., 2014). These findings are also in agreement with proposals from mathematical models suggesting that a ‘fixed’ hybrid E/M state is certainly sufficient to promote metastatic progression (Hong *et al*., 2015; Jolly *et al*., 2016). Whether these observations can be extended to human tumors awaits the characterization of markers accurately defining the E/M status. In contrast to these proposals, other studies favor that switching across the spectrum of different cell phenotypes underlying dynamic and plastic E/M states might be indeed advantageous for metastatic progression. Intriguingly, a gene expression signature derived from E6.5 mouse embryos, representing a high cellular plasticity state due to embryonic spatiotemporal cellular dynamics, predicts the metastatic behavior of breast cancer cells and has prognostic value in the clinical setting (Soundararajan *et al*., 2015).

Although cell tracing experiments along with intravital imaging have provided further support for the occurrence of EMT process *in vivo*, additional interrogations remain such as whether the plasticity processes are relevant for all tumor subtypes. Due to the limited information attained from available *in vivo* models, so far restricted to specific carcinoma mouse models (SCC, breast, pancreas, CRC), generalizations cannot be made. Indeed, some studies support that both plasticity‐dependent and plasticity‐independent mechanisms can operate in different cancer contexts (Brabletz, 2012b; Diepenbruck and Christofori, 2016; Somarelli *et al*., 2016). Therefore, additional studies using innovative genetically engineered mouse models to reliable trace and analyze tumor cells responsible for seed metastasis, together with implementation of higher‐resolution *in vivo* intravital imaging microscopy, would certainly contribute to better understand the biological relevance of EMT and plasticity processes to metastasis in different tumor contexts.

## 3 EMT in human tumors: translation to clinical diagnosis and prognosis

Despite the accumulated evidence on EMT involvement in metastasis supported by recent evidences from preclinical models, its translation into the clinical setting is still challenging. This is not only due to the difficulty to ascertain EMT in pathological analyses of human biopsies (Tarin *et al*., 2005) but also to the heterogeneity inherent to tumors, the diverse metastatic behavior associated with different cancer contexts and the leap that entails translating results from homogenous cell lines in culture and preclinical models to tumors developed within persons. Moreover, the dynamic and transient nature of EMT comprising a broad spectrum of intermediate phenotypes, which might be represented in the primary tumor at spatially and temporarily distinct ratios (Lambert *et al*., 2017; Nieto *et al*., 2016), adds further complexity to the use of tumor EMT status as an indicator of diagnosis and/or prognosis. Thus, one of the pending challenges is the characterization of a few number of genes or proteins that could be studied in human samples to predict the establishment or acquisition of EMT or hybrid E/M features, even at early tumor stages, along with the detection of the reverse MET process linked to the micro‐ to macrometastasis formation (Lambert *et al*., 2017; Nieto *et al*., 2016). In this regard, upon intense efforts, several groups have focused on the identification of genetic or protein EMT signatures able to differentiate the epithelial versus mesenchymal phenotypes in cancer cells (Pasquier *et al*., 2015; Steinestel *et al*., 2014; Zeisberg and Neilson, 2009).

### 3.1 Detection of EMT markers in tumor samples

Since the initial characterization of the hallmark markers linked to the EMT process (Kalluri and Weinberg, 2009; Moreno‐Bueno *et al*., 2009; Thiery, 2003; Thiery *et al*., 2009), changes in the expression of several EMT‐associated genes and/or proteins have been used to assess EMT status in human tumor samples in an effort to establish an association with clinical significance (Pasquier *et al*., 2015; Steinestel *et al*., 2014). However, this is still a debated question among pathologists being some of them reluctant to accept the biological significance of the EMT process in tumor development (Tarin *et al*., 2005). In fact, cells that have undergone EMT are not frequently observed in tumor samples probably because biopsies represent a precise moment during tumor development, whereas cells suffering EMT could be shifting among hybrid E/M states and appear at different time points during tumorigenesis. Additionally, cells acquiring a full mesenchymal phenotype (endpoint EMT) would resemble stromal cells surrounding the tumor, making its clinical assessment by conventional histopathological techniques difficult (Ledford, 2011; Tarin *et al*., 2005). Indeed, the expression of some EMT‐TFs, such as Snail1, has been detected not only in tumor cells but also in activated stromal fibroblasts favoring invasiveness and metastasis (Alba‐Castellon *et al*., 2016; Franci *et al*., 2006; Rowe *et al*., 2009), adding complexity to the histological determination of EMT status based on single EMT markers.

Although the number of studies that include tumor analyses of EMT markers is considerable, most common alterations studied within human tumor samples are related to the loss or aberrant expression of proteins required to maintain the epithelial phenotype and usually involved in cell–cell adhesion (Steinestel *et al*., 2014), being E‐cadherin one of the strongest markers routinely used in the clinic for cancer diagnosis or progression (Pasquier *et al*., 2015). E‐cadherin downregulation is considered a hallmark of EMT together with the concomitant overexpression of specific mesenchymal markers such as N‐cadherin and vimentin, among others (Kalluri and Weinberg, 2009; Moreno‐Bueno *et al*., 2009; Nieto, 2011; Thiery, 2002; Thiery and Sleeman, 2006; Yang and Weinberg, 2008). Apart from diffuse gastric and lobular breast carcinomas where germline mutations and somatic inactivation of the E‐cadherin (*CDH1*) locus occurs (Berx *et al*., 1996; Fitzgerald and Caldas, 2004; Guilford *et al*., 1998; Peinado *et al*., 2004), different studies have supported E‐cadherin detection as a diagnostic marker in several tumor types. Immunohistochemically detected E‐cadherin status correlates with the differentiation grade and histological type in breast carcinomas (Acs *et al*., 2001; Gamallo *et al*., 1993; Moll *et al*., 1993) and E‐cadherin expression is also used for the differential diagnosis of papillary thyroid carcinoma (Ceyran *et al*., 2015). Besides these tumors, where E‐cadherin status supports diagnosis, its aberrant expression has also been associated with diverse clinicopathological features in various carcinomas such as colon, lung, ovary, esophagus, prostate, or cervix, among others (reviewed in Steinestel *et al*., 2014). However, some studies indicate that N‐cadherin overexpression, rather than E‐cadherin loss, is a predictive marker of lymph node metastasis in gastric tumors (Okubo *et al*., 2017) as well as a promoter of thyroid tumorigenesis (Da *et al*., 2017).

Considering that loss of cell–cell adhesion and apico‐basal polarity are hallmarks of EMT, the alterations in key molecular players conferring those cell properties, apart from cadherins, have also been used as markers of EMT in tumor samples. The relocalization to the cytoplasm and/or nucleus of β‐catenin, an architectural membrane protein in adherens junctions, has been associated with CRC progression (reviewed in Schmalhofer *et al*., 2009) and similar relocalization of p120 catenin has been detected in breast tumors and carcinosarcomas (Sarrio *et al*., 2004, 2008). In this line, downregulation or loss of function of proteins involved in epithelial homeostasis such as tight junction components [claudins, occludins, ZO‐1 (zonula occludens‐1)] is frequently observed in several carcinomas (Steinestel *et al*., 2014; Zeisberg and Neilson, 2009), whereas the expression shift from epithelial keratins to mesenchymal vimentin, required for cell migration and invasion, is also commonly assessed in diverse types of tumors (Sarrio *et al*., 2008; Satelli and Li, 2011; Zeisberg and Neilson, 2009). Some specific protein signatures including few epithelial and mesenchymal markers have also been reported. In this regard, a study with 29 markers in around 500 breast tumors revealed that EMT‐related proteins were normally overexpressed in basal‐like breast carcinomas and in carcinosarcomas (Sarrio *et al*., 2008), although its diagnostic potential remains to be explored. In addition, one study of four genes [E‐cadherin, MMP9 (matrix metalloproteinase‐9), TCF3 (transcription factor 3)/E47 and ID2 (inhibitor of differentiation 2)] has validated their prognostic value, in terms of overall survival, in a large cohort of hepatocellular carcinomas (HCC) (Kim *et al*., 2010). Besides, deregulated expression of canonical EMT‐TFs has been associated with several tumor types, especially those displaying EMT such as breast, cervix, ovary, or colon (reviewed in De Craene and Berx, 2013; Peinado *et al*., 2007; Polyak and Weinberg, 2009; Steinestel *et al*., 2014). Furthermore, Snail1 nuclear expression was found in invasive ductal carcinomas (Blanco *et al*., 2002) and in basal‐like breast tumors contexts (Becker *et al*., 2007; Geradts *et al*., 2011) and correlated with lymph and cervical node and distant metastasis of breast and head and neck squamous cell carcinoma (HNSCC) (Yang *et al*., 2007), or even in stromal cells close to invasive areas (Franci *et al*., 2006). Also, ZEB1 expression was associated with prostate cancer (PCa) progression and metastasis (Putzke *et al*., 2011), while Twist1 plus ZEB2 expression was related to early disease recurrence in HCC (Yamada *et al*., 2014). However, the individual protein status of Snail1, Twist1, or ZEB1 is not regularly used for diagnostic purposes, whereas, as discussed later, their expression is most commonly linked to patient outcome and/or therapy resistance (Nieto *et al*., 2016; Pasquier *et al*., 2015; Steinestel *et al*., 2014; Yeung and Yang, 2017). Recently, one score determination of EMT‐related splicing factors was also proposed as an EMT index with potential prognostic value in a small sample of breast carcinomas (Fici *et al*., 2017), although its clinical application awaits further studies in larger series and additional tumor types. Overall, there are still serious concerns on the clinical use of the status of most EMT‐TFs plus other countless EMT markers that have been also correlated with prognosis (reviewed in Malek *et al*., 2017; Steinestel *et al*., 2014).

### 3.2 EMT gene signatures and their clinical application

Many clinical studies have demonstrated that distinct EMT signatures predict poor prognosis, tumor aggressiveness, and, in general, worse patient outcome in different types of cancer (Steinestel *et al*., 2014; Yeung and Yang, 2017). However, a review of the literature suggests that there are caveats in the numerous reported associations of EMT gene signatures and prognosis, reducing their clinical impact. Besides, discrepancies establishing a link between EMT gene profiles and overall survival in different types of tumors have been reported as well (Steinestel *et al*., 2014; Taube *et al*., 2010). The underlying cause for these controversies may arise from the fact that some EMT gene signatures were deduced from cell lines and their impact on tumor samples is thus limited.

Additionally, there is ample variability in the published studies regarding sample size, clinical evaluation of patients, methods used to assess the expression of EMT‐associated markers, and cutoff values established, rendering unreliable prognostic information in many cases (Pasquier *et al*., 2015). The fact that clinical samples are obtained from tumors with inherent heterogeneity in which hybrid E/M states are expected to be restricted in time and space to specific tumor areas as well as influenced by the tumor microenvironment just adds another layer of difficulty to translate EMT signatures to patients’ prognosis. In spite of this, several EMT gene signatures support tumor subtype classification linked to clinical outcome in different types of cancer (Pasquier *et al*., 2015; Steinestel *et al*., 2014; Yeung and Yang, 2017). For instance, in breast cancer, the basal‐like and claudin‐low subtypes, characterized by poor prognosis, present a clear EMT signature (Hennessy *et al*., 2009; Sarrio *et al*., 2008; Taube *et al*., 2010). As expected, most downregulated genes found in those profiles were classified as epithelial markers involved in cell–cell adhesion and apico‐basolateral polarity, including E‐cadherin, desmosomal [DSG3 (desmoglein 3), DSP (desmoplakin)], and tight junction components [CLDN4/7 (claudin 4/7)] among others, whereas mesenchymal genes such as vimentin and MMP2/9 were upregulated (Chui, 2013). These EMT‐related gene signatures denoted changes in the expression levels of genes involved in signaling pathways (reviewed in De Craene and Berx, 2013; Lamouille *et al*., 2014). In general, these gene expression changes favor the activation of intracellular signaling cascades related to tumor progression (Garg, 2013), reflecting that many regulatory networks are dynamically orchestrated during EMT (De Craene and Berx, 2013; Nieto and Cano, 2012).

The extensive data generated from different EMT gene signatures have not helped to clarify whether this information is clinically sound. However, new meta‐analyses are starting to shed some light regarding this important issue. A recent meta‐analysis in metastatic breast cancer including 3218 patients has revealed that the individual or combined high expression levels of the EMT‐TFs, Twist1, Snail1, and Snail2, significantly correlated with poor prognosis (Imani *et al*., 2016). Another meta‐analysis links high Twist1 or Snail1 expression with poor prognosis related to all clinical outcomes in various carcinomas such as lung and gastrointestinal tumors (Zhang *et al*., 2014a). In an effort to quantify the relationship between EMT and cancer progression, Tan *et al*. (2014) established an EMT scoring method based on EMT gene signatures obtained from ovarian, breast, bladder, lung, colorectal, and gastric carcinomas (Tan *et al*., 2014). When this EMT score was applied to different tumor types, it unveiled correlations between EMT status and poorer survival in ovarian and colorectal cancer, but not in breast carcinomas, and allowed to establish a connection with chemotherapeutic resistance. A recent TCGA pan‐cancer analysis of EMT markers in 10 244 tumor mRNA samples representing 32 different types of cancer established a 16‐gene EMT signature significantly associated with worse outcome in all tumor types (Gibbons and Creighton, 2017).

In addition to the identification of recurrent EMT profiles in different tumor contexts, the characterization of molecular gene signatures in specific tumors has been associated with cell migration, development, and/or stemness, among other aspects, and might be of clinical translational potential (Serrano‐Gomez *et al*., 2016). Indeed, these specific signatures could be used as new tools for understanding cell plasticity in tumor biopsies as well as for diagnosis and prognosis (Schoenhals *et al*., 2009). In this sense, it is noteworthy to mention that the gene signature obtained from E6.5 embryonic development, denoting a gene expression pattern associated with considerable cellular plasticity, shows a higher significant predictive value in terms of relapse and distant metastasis‐free survival in comparison with gene signatures associated with the expression of several EMT‐TFs (Soundararajan *et al*., 2015). Nonetheless, the large number of genes present in the E6.5 signature precludes its straight application to tumor biopsies. The refinement of such innovative gene signatures might benefit the clinical translation of tumor EMT status in the near future.

Besides, EMT‐miRNA gene signatures have been associated with specific tumor contexts (reviewed in Diaz‐Lopez *et al*., 2014; Trager and Dhayat, 2017). The miR‐200 family (miR‐200f), composed of tumor suppressors involved in EMT inhibition and apoptosis or proliferation (Brabletz and Brabletz, 2010; Feng *et al*., 2014), has been extensively characterized. In the context of human tumors, decreased expression of the miR‐200f has been detected in many cancer types such as breast, CRC, HCC, non‐small‐cell lung cancer (NSCLC), renal clear cell carcinoma, and PCa (reviewed in Zaravinos, 2015). Additionally, in endometrial carcinosarcomas, miR‐200f expression is restricted to the carcinoma area, but absent in the sarcomatous regions (Castilla *et al*., 2011), validating the key role of miR‐200 members in maintaining the epithelial phenotype *in vivo*. Moreover, a link between miRNA signatures and expression of specific EMT‐TFs has also been described in carcinosarcomas (Diaz‐Martin *et al*., 2014) and PCa (Sekhon *et al*., 2016). Significantly, in experimental cancer models, the upregulation of miR‐200f influences the cancer cell secretome favoring metastasis associated with the reacquisition of epithelial traits (Korpal *et al*., 2011).

Although the latter findings were validated by clinical correlations, the actual utility for diagnosis and/or prognosis is still to come. Additional efforts integrating mRNA and miRNA signatures related to EMT and epithelial plasticity in different tumor contexts are required to further translate this knowledge to the clinical context.

### 3.3 Translational potential of EMT to liquid biopsy

A specific consideration requires the analysis of EMT features in liquid biopsies, particularly in circulating tumor cells (CTCs), currently being characterized and emerging as a new promising prognostic factor in different types of tumors (reviewed in Cabel *et al*., 2017). In fact, CTC detection, to different thresholds depending on the tumor type, is indicative of a poor prognosis linked to the ability to seed metastasis from the primary tumor, as in endometrial, HCC, NSCLC, CCR, and breast tumors, among others (Alonso‐Alconada *et al*., 2014; Barbazan *et al*., 2014; Bidard *et al*., 2014; Cristofanilli *et al*., 2004; Janni *et al*., 2016; Li *et al*., 2013; Wang *et al*., 2013; Wu *et al*., 2015; Yu *et al*., 2013). Analyses of CTCs from patients have also provided a strong link between EMT and metastasis in the clinical setting (Nieto *et al*., 2016). CTCs from a wide range of tumor types are characterized by the expression of both epithelial and mesenchymal markers (Alonso‐Alconada *et al*., 2014; Yu *et al*., 2013), indicative of EMT plasticity. The label‐free isolation of CTCs in patient samples has also allowed the identification of clusters of CTCs apparently entailing higher metastatic potential than single CTCs (Aceto *et al*., 2014; Cheung *et al*., 2016; Sarioglu *et al*., 2015). These CTC clusters may represent collective migration of cells, some of which retain epithelial characteristics endowing phenotypic advantage to rapidly grow macrometastasis at target organs (Diepenbruck and Christofori, 2016). Alternatively, CTC clusters can correspond to the association of individually seeded CTCs together with blood platelets that are indeed emerging as key regulators of EMT and tumor cell survival during their blood dissemination (Labelle *et al*., 2011; Leblanc and Peyruchaud, 2016; Takemoto *et al*., 2017) in addition to potential therapeutic targets (Li *et al*., 2016a,b; Roop *et al*., 2013).

Nevertheless, the assessment of the actual relevance of CTC clusters in the clinical scenario requires further evaluation in larger series of samples and different stages of disease progression. Although the presence of an EMT and/or hybrid E/M phenotypes in CTCs has been correlated with several factors reflecting worse patient outcome (Nieto *et al*., 2016), there is again debate over the assay allowing the detection of CTCs, which are expected to have high epithelial/mesenchymal plasticity (Alix‐Panabieres *et al*., 2017; Liu *et al*., 2014). Thus, the main obstacle for the clinical application of CTC detection is that CTCs are presently isolated using microfluidic separation reliant on EpCAM, an epithelial marker (Harouaka *et al*., 2014) whose expression is completely lost when tumor cells begin to express key mesenchymal markers such as N‐cadherin and/or vimentin (Armstrong *et al*., 2011). Considering these aspects, EpCAM‐based detection seems to be insufficient for characterizing the CTC population. In this regard, the implementation of new immunostaining methods based on a combination of different epithelial and mesenchymal biomarkers has emerged as the current best approach for CTC detection (Harouaka *et al*., 2014) improving the capture of cells undergoing hybrid E/M states (Barriere *et al*., 2014; Parisi *et al*., 2016). Additional parameters such as size, deformability, and other physical properties should also be incorporated for advanced detection of CTCs with plastic phenotypes (Alix‐Panabieres *et al*., 2017).

## 4 Current clinical status of EMT

### 4.1 EMT application to the prediction of therapy resistance

EMT has been strongly related to two additional properties of disseminating tumor cells, most likely intrinsically related, the acquisition of stemness properties (Lambert *et al*., 2017; Nieto *et al*., 2016) and of therapy resistance (Smith and Bhowmick, 2016). Accumulated evidence has lent support to the link between EMT and stemness, implying an association between EMT and tumor‐ or metastasis‐initiating capabilities either in cell lines or in different types of tumors (Brabletz, 2012a; Mani *et al*., 2008; Morel *et al*., 2008). Further studies in *in vivo* breast cancer mouse models corroborated Snail1 expression associated with the acquisition of stem‐like features in neoplastic cells (Ye *et al*., 2015). On the other hand, several studies support that EMT induction confers resistance to chemo‐ and radiotherapeutic treatments (Gupta *et al*., 2009; Kajita *et al*., 2004; Kurrey *et al*., 2009; Perez‐Losada *et al*., 2003) as also reinforced with the preclinical models discussed above (Fischer *et al*., 2015; Zheng *et al*., 2015). However, the present knowledge does not support that EMT association with resistance is necessarily linked to cell stemness. While acquired resistance has been progressively connected to EMT and more specifically, the increase in some EMT‐TFs has been related to a reduction in the therapeutical response in several preclinical models (Du and Shim, 2016), the link between EMT and cell stemness properties is not as clear (Brabletz, 2012a). Several recent studies have depicted distinct situations in which uncoupling EMT from stem cell‐like properties is observed. This is the case for PRRX1 whose expression in breast carcinoma cells inhibits stemness, while its silencing, leading to MET, is required for metastasis outgrowth associated with stem‐like properties (Ocaña *et al*., 2012). The opposite is true for Twist1, and EMT‐TF inducing cell stemness (Mani *et al*., 2008), whose expression should be turned off at distant sites to allow metastatic growth in breast and SCC tumors (Ocaña *et al*., 2012; Tsai *et al*., 2012). Interestingly, Twist1 silencing in breast cancer cells can be mediated by ID1 (inhibitor of differentiation 1) promoting a stem‐like phenotype while maintaining epithelial properties at distant sites (Stankic *et al*., 2013). Uncoupling EMT and stem cell‐like potential linked to metastasis has also been described in prostate and bladder cancer cell models (Celia‐Terrassa *et al*., 2012), even if the detailed involvement of individual EMT‐TFs has not yet been defined. Besides, recent data from *in vivo* breast cancer mouse models also support that metastatic outgrowth (i.e., metastasis‐enhancing stem cell capacity) might be independent of epithelial plasticity (Beerling *et al*., 2016), adding further complexity to the current situation. Overall, the present knowledge suggests that EMT and stemness might not be necessarily coupled, and this association might depend on specific EMT‐TFs and the type of tumor (Brabletz, 2012a,b; Celia‐Terrassa and Kang, 2016; Nieto, 2013).

Regarding therapeutic resistance, increasing evidence supports the association of EMT with chemoresistance, particularly favoring the multidrug resistance (MDR) phenotype (da Fonseca *et al*., 2016) but also with radioresistance (Smith and Bhowmick, 2016). Lately, numerous studies have tried to comprehend the resistance mechanisms in cells undergoing EMT in several tumor types (reviewed in Du and Shim, 2016; Smith and Bhowmick, 2016) in addition to the development of targeted therapeutic strategies to halt the EMT process based on blocking directly or indirectly specific related pathways (Du and Shim, 2016; da Fonseca *et al*., 2016). However, most of these studies have only revealed the low efficiency of these treatments, probably associated with the existence of alternative pathways able to induce and/or regulate the transient and plastic EMT phenotype (Zhou *et al*., 2017).

**Figure 2:**
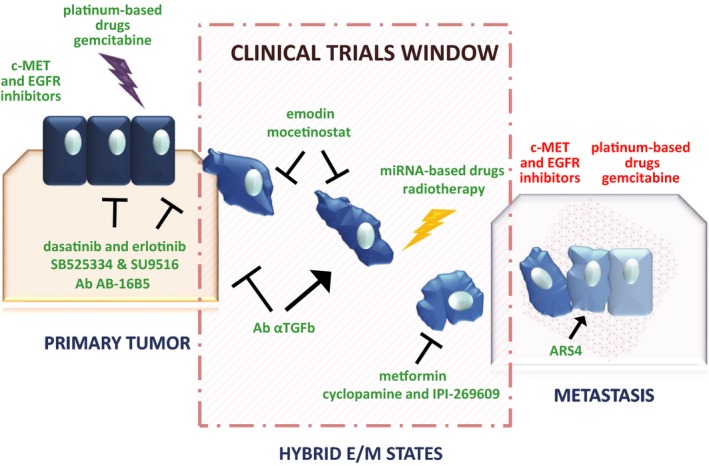
Illustration representing several anti‐EMT therapies targeting epithelial plasticity during metastasis. The potential action of several drugs regarding EMT, hybrid E/M, or MET status during tumor progression is shown. Both standard treatments and drugs under current clinical testing are included. Sensitivity (green) or resistance (red) to the indicated treatments during the course of metastasis is associated with cellular plasticity state. Based on the present knowledge, the best opportunities for therapeutic success seem to rely on targeting the hybrid E/M states during cancer cell metastasis.

The first report linking EMT to resistance dates from 1996, when specific antibodies against TGF‐β, one of the strongest EMT inducers, restored drug sensitivity in resistant tumors to alkylating compounds (Teicher *et al*., 1996) (Fig. 2). Up until now increasing findings support that EMT favors resistance (reviewed in Du and Shim, 2016) although the underlying molecular mechanisms remain partly unsolved. Indeed, a study of specific therapeutic agents in different preclinical models has revealed that increased expression levels of some EMT‐TFs are strongly associated with a low therapy response rate (Du and Shim, 2016). To mention some of them, Snail1 expression has been related to cisplatin and 5‐fluorouracil resistance in HNSCC and in NSCLC (Hsu *et al*., 2010) along with breast tumor cells (Zhang *et al*., 2012), respectively. Furthermore, cells undergoing EMT overexpress some specific ABC transporters (da Fonseca *et al*., 2016) involved in resistance mechanisms. In fact, the promoters of ABC transporters contain several binding sites for EMT‐TFs (Saxena *et al*., 2011). Moreover, the resistance phenotype observed in NSCLC patients treated with c‐MET and EGFR (EGF receptor) tyrosine kinase inhibitors is associated with ZEB1 expression (Della Corte *et al*., 2015; Rastogi *et al*., 2016). Noticeably, in lung cancer cells in which treatment with EGFR inhibitors induced EMT, a differential sensitivity between epithelial and mesenchymal cells was observed (Yauch *et al*., 2005). On the other hand, an association with EMT has also been reported in gemcitabine‐resistant highly invasive PCa cells, in platinum‐resistant CCR cells as well as in post‐ionizing radiation‐associated metastasis in patients with advanced lung cancer (Du and Shim, 2016).

Finally, regarding liquid biopsy, CTC isolation and characterization have been recently addressed as a straightforward method to evaluate clinical response (Alix‐Panabieres *et al*., 2017). Patients with refractory breast cancer presented higher levels of mesenchymal CTCs (Yu *et al*., 2013), while CTC changes during chemotherapy treatment were significantly associated with progression‐free survival and overall survival. These facts support CTC‐based models as prognostic tools for considering therapy, and/or to stratify patients and adjust therapeutic factors in clinical trials (Bidard *et al*., 2014). These results support the clinical application of the characterization of EMT‐associated markers in patient‐derived CTCs to predict treatment response (McInnes *et al*., 2015).

### 4.2 Are anti‐EMT therapies feasible?

Although much interest was raised in the past decade in regard to EMT as a potential therapeutic target, the development of specific novel drugs against EMT or EMT‐related signaling pathways constitute a tremendous challenge of current oncology and at present there are only a few studies based on treatments directly targeting EMT (Fig. 2).

An example is the inhibition of ILK, an integrin‐linked kinase involved in AKT pathway activation leading to EMT (Jiang *et al*., 2015). Treatment with emodin (1,3,8‐trihydroxy‐6‐methylanthraquinone) (Bruney *et al*., 2016), promotes a MET process by targeting ILK in ovarian cancer (Lu *et al*., 2016) and endometrial sarcoma cells (Zheng *et al*., 2016), in addition to reducing EMT through the ILK/AKT/mTOR signaling pathway in breast cancer cells (Ma *et al*., 2016) (Fig. 2). Besides, it has been proposed that emodin is able to inhibit Twist1‐depended EMT in HNSCC cells by inhibiting the WNT/β‐catenin and AKT pathways (Way *et al*., 2014). Interestingly, an artemisinin–melphalan conjugate drug (ARS4, an antimalarial agent) is highly toxic in ovarian cancer cells, but not in normal cells (Li *et al*., 2016c). In this context, ARS4 induced phenotypic changes resembling a MET process and promoted cell cycle arrest and apoptosis (Li *et al*., 2016c) (Fig. 2). Additionally, the small molecule cyclopamine, an steroidal alkaloid, and its semisynthetic analogue, IPI‐269609, have shown efficacy against pancreatic cancer metastasis through the inhibition of Hedgehog signaling, downregulation of Snail1, and upregulation of E‐cadherin in cells undergoing EMT (Katoh and Katoh, 2009). More recently, metformin (1,1‐dimethylbiguanide hydrochloride), a well‐tolerated treatment for type 2 diabetes mellitus which inhibits hepatic glucose production while increasing glucose uptake as well as reducing insulin resistance in peripheral tissue and gluconeogenesis (Goodwin *et al*., 2009; Hundal *et al*., 1992), has shown promise in cancer treatment (Barriere *et al*., 2013). In combination with chemotherapeutic agents, metformin seemed effective against PCa cell viability by repressing vimentin and N‐cadherin while inducing E‐cadherin expression (Zhang *et al*., 2014b) (Fig. 2).

Interestingly, the emergence of targeted therapies against signaling regulators of EMT might lead toward clinical benefits due to the specific targeting of cancer cells undergoing EMT. In this line, dasatinib, a SRC kinase inhibitor, decreased lung cancer cell growth upon EMT induction (Wilson *et al*., 2014). Additionally, the combination of dasatinib with erlotinib, another SRC inhibitor, has emerged as a promising treatment to prevent EMT‐induced resistance (Sesumi *et al*., 2017) (Fig. 2). Moreover, high‐throughput screenings aimed at identifying anti‐EMT‐related drugs have been recently developed (Arai *et al*., 2016; Carmody *et al*., 2012; Gupta *et al*., 2009). In one of them, 19 compounds were initially chosen as potential EMT and stemness inhibitors, uncovering a synthetic compound, ML245 (BRD‐K59019422‐001‐01‐3), that restrained cancer cell progression although additional studies are necessary to clarify the exact mechanism of action (Carmody *et al*., 2011). Another massive screening targeted toward specific cancer cell states and stemness properties identified salinomycin, a potassium ionophore, with potential effects on undifferentiated breast carcinoma cells (Gupta *et al*., 2009). Interestingly, two additional drugs, SB525334 and SU9516, targeting TGFβR1 (TGFβ receptor 1) and CDK2 (cyclin dependent kinase 2), respectively (Fig. 2), were identified in another high‐throughput screening showing EMT inhibitory activity in lung cancer cells (Arai *et al*., 2016).

On the other hand, recent therapeutic strategies have been focused on EMT targeting through RNA interference, miRNA, and agomiRs/antagomiRs. However, their clinical application is still very limited due to unresolved issues such as target organ delivery and immune response circumvention (Shah and Calin, 2014). Despite these disadvantages, several studies showed their success in the downregulation of specific EMT mediators, advocating for their use as novel therapies or as sensitizers to radio‐ or chemotherapies able to block not only the EMT process but also metastasis (de Jong *et al*., 2015; Smith and Bhowmick, 2016) (Fig. 2). Accordingly, EMT might be neutralized using miR‐875‐5p, which mediates EGFR downregulation in PCa cells, promoting a radiosensitizing effect (El Bezawy *et al*., 2017). Additionally, the inhibition of VEGFR (VEGF receptor) expression using three artificial miRNA in PCa cells and xenograft mouse models showed a synergistic effect with standard chemotherapy and was associated with EMT inhibition (Huang *et al*., 2017). Indeed, it has been recently described that the combination of vaccines with miR‐200 agomiRs or shZEB1 significantly inhibited EMT features in melanoma cells and prompted an immune response that blocked melanoma growth and metastasis in mouse models (Wang *et al*., 2016). Recent advances in the delivery of oligonucleotides, approved by the FDA for other pathologies like several nervous system disorders (Khorkova and Wahlestedt, 2017), pave the way for future advances in the application of oligonucleotide‐based therapies targeting EMT and metastasis.

Finally, based on the epigenetic regulation of EMT (reviewed in Serrano‐Gomez *et al*., 2016), it has been postulated that selected EMT‐targeting epigenetic drugs can be used alone or in combination with conventional chemotherapies to overcome drug resistance (Sun and Fang, 2016). In this line, specific epigenetic inhibitors, such as sorafenib or mocetinostat, could also serve as powerful tools to target epigenetic pathways regulating EMT *in vivo* (Kiesslich *et al*., 2013) (Fig. 2). Sorafenib reverses histone modifications related to EMT in lung carcinoma cells (Zhang *et al*., 2013), and mocetinostat is a histone deacetylase (HDAC) inhibitor able to reverse the EMT phenotype and sensitize resistant PCa cells to docetaxel by restoring miR**‐**203 expression and promoting ZEB1 inhibition (Meidhof *et al*., 2015).

### 4.3 Clinical trials for EMT inhibition

Recent reviews have summarized the current status of clinical studies designed to understand the role of specific EMT suppressor treatments (reviewed in Chaffer *et al*., 2016; Marcucci *et al*., 2016). Actual advances for targeted anti‐EMT therapies to the clinical setting obviously require the development of clinical trials. Currently, there are some randomized clinical studies (https://clinicaltrials.gov/) in which one of the main selection criteria is the expression of specific EMT markers and the final aim is the direct or indirect EMT inhibition using novel molecular‐based personalized therapies (Table 1). Most of these clinical trials are focused on testing the effects of different therapies on CTCs displaying specific EMT features. This is the case for the clinical trial based on the aspirin treatment for metastatic breast and colorectal CTCs with EMT features (NCT02602938, clinical trial identifier) (Table 1). Interestingly, previous analyses reported the effectiveness of aspirin as a chemoprevention agent in some tumor types (Santilli *et al*., 2016). Other clinical trials focus on isolation and detection methods of CTCs based on EMT markers (Table 1).

**Table 1:** Active EMT‐related clinical trials currently in patient recruitment phase^a^

ID^b^	Title	EMT‐related target
NCT02412462	Phase I Dose Escalation Study of AB‐16B5 in Subjects With an Advanced Solid Malignancy	Secreted clusterin (sCLU)
NCT02913859	Hormone Therapy With or Without Definitive Radiotherapy in Metastatic Prostate Cancer	N‐cadherin, E‐cadherin, vimentin^c^
NCT02602938	Aspirin on CTCs of Advanced Breast and Colorectal Cancer (ACABC)	Number and subtype of CTCs
NCT01990196	Neoadjuvant Phase II Study Comparing the Effects of AR Inhibition With/Without SRC or MEK Inhibition in Prostate Cancer	N‐cadherin and vimentin expression
NCT02119559	Circulating Tumor Cells as Early Predictive in Head‐and‐Neck Squamous‐Cell Carcinoma (CIRCUTEC)	CTCs on the progression‐free survival and EMT markers^d^
NCT02119559	Isolation of Circulating Tumor Cells Using a Novel EMT‐Based Capture Method (CTC‐EMT)	Presence of EMT markers on the prognosis^d^
NCT02951897	Application of Detecting Circulating Tumor Cells in the Accurate Treatment of Early Stage Lung Adenocarcinoma (CTCs detection)	Characterization of epithelial (E) CTCs, mesenchymal (M) CTCs, and epithelial/mesenchymal (E/M) CTCs in early diagnosis^d^

a Status March 2017.

b ID: identifier study number in clinical trials page: https://clinicaltrials.gov/.

c Analysis of EMT markers (N‐cadherin, E‐cadherin, vimentin) before radiotherapy or after radiotherapy to establish disease progression‐free survival.

EMT markers nonspecified.

d EMT markers: cytokeratins 8, 18, and 19, EpCAM, vimentin, and TWIST.

Ongoing EMT‐related clinical trials also include the use of known cancer targeted therapies, immunotherapies, and/or novel compounds. An example of conventional cancer therapy is the randomized phase II assay in patients with PCa (NCT01990196) using hormonotherapy (androgen receptor inhibition) with and without chemotherapy against SRC or MEK (degarelix, enzalutamide, trametinib, or dasatinib) in terms of EMT inhibition (Table 1). Regarding therapies based on the use of antibodies, a phase I clinical trial with humanized monoclonal antibody AB‐16B5 (NCT02412462) in advanced solid tumors is ongoing (Table 1 and Fig. 2). AB‐16B5 was previously characterized for the inhibition of the potent EMT inducer secretory clusterin (sCLU) (Tremblay *et al*., 2010), a stress‐activated and apoptosis‐associated chaperone that protects cells from various stresses (Shiota *et al*., 2012). Moreover, sCLU was also significantly upregulated during tumor progression and metastasis (Wang *et al*., 2012). Finally, other clinical studies are focused on novel compounds as potential EMT‐interfering drugs. MK‐0646 or dalotuzumab, a humanized IgG1 monoclonal antibody which binds and blocks IGF1R (IGF1 receptor) (Atzori *et al*., 2011), is evaluated in combination with gemcitabine, or gemcitabine plus erlotinib, in patients with advanced pancreatic cancer to test its role in progression‐free and overall survival in correlation with the expression of IGFIR, AKT, and diverse EMT biomarkers (NCT00769483) (Table 1).

Already finalized clinical trials have provided valuable data confirming the potential benefit of the treatments aimed at targeting EMT. This is the case of the phase I trial performed in advanced solid tumor patients that demonstrated the effectiveness of MRX34 (Beg *et al*., 2017), a liposomal miR‐34a mimic, which inhibits Snail1‐mediated EMT and the NOTCH pathway in pancreatic cancer cell lines (Tang *et al*., 2017).

Despite the advances in the molecular understanding of EMT and cell plasticity processes, not only regarding invasiveness and metastasis but also in association with therapy response and stem cell‐like properties, further experimental work is necessary to render EMT knowledge beneficial for clinical standard of care. An important word of caution toward therapeutic approaches aimed at blocking EMT relates to the inherent plasticity of the EMT process during tumor progression and metastasis. While some therapies could help halt tumor cell invasion and dissemination, they might be highly deleterious for metastasis outgrowth by favoring the MET program. In addition, they may not be exploited for targeting early disseminated tumor cells believed to shred from primary tumors before diagnosis. From the present knowledge, targeting the hybrid E/M phenotype (Fig. 2) seems the most promising strategy for the clinical application of EMT and epithelial plasticity in the, hopefully, near future.

## 5 Concluding remarks and future perspectives

The EMT field has experienced an enormous progress in the last years leading to a comprehensive understanding of the molecular pathways and regulators of the process. In the context of metastasis, the demonstration of the transient and reversible nature of EMT process, the requirement of MET for metastasis outgrowth along with the report of hybrid E/M states during the metastatic cascade have been key advances. Preclinical genetic mouse models and innovative intravital imaging have also helped to demonstrate the plasticity of the process *in vivo* and highlighted the nonredundant and complementary function**s** of EMT‐TFs and their specificity in distinct tumors. All these recent studies have contributed to clarify the key *in vivo* role of epithelial plasticity processes at different metastasis steps. Despite all this progress, EMT clinical translation is still very limited. Nevertheless, support for using EMT markers and/or EMT signatures as predictor or prognosis factors is continuously increasing. This is particularly relevant in relation to acquired resistance for which further improvements in CTC detection methods aimed at capturing different plastic E/M states are envisioned for the near future. In addition, better understanding of the contribution of the stromal component, in particular activated fibroblast and immune components, to modulate epithelial plasticity processes, will undoubtedly contribute to improve its translation potential.

It is anticipated that the continuous development of highly sophisticated preclinical models and novel imaging techniques, together with innovative high‐throughput screening platforms to target hybrid E/M phenotypes, will provide new avenues to explore the clinical potential of epithelial plasticity in the near future.
